# Screening and verification of differentially expressed serum proteins in children with severe adenovirus pneumonia

**DOI:** 10.3389/fpubh.2024.1476330

**Published:** 2024-11-20

**Authors:** Zheng-Xiang Gao, Hong Xu, Qu Yang, Liang Xie, Li-Na Chen, Han-Min Liu

**Affiliations:** ^1^Department of Laboratory Medicine, West China Second University Hospital, Sichuan University, Chengdu, China; ^2^Key Laboratory of Birth Defects and Related Diseases of Women and Children (Sichuan University), Ministry of Education, Chengdu, China; ^3^NHC Key Laboratory of Chronobiology (Sichuan University), Chengdu, China; ^4^The Joint Laboratory for Lung Development and Related Diseases of West China Second University Hospital, Sichuan University and School of Life Sciences of Fudan University, West China Institute of Women and Children's Health, West China Second University Hospital, Sichuan University, Chengdu, China; ^5^College of Laboratory Medicine, North Sichuan Medical College, Nanchong, China; ^6^Department of Pediatric Pulmonology and Immunology, West China Second University Hospital, Sichuan University, Chengdu, China; ^7^Sichuan University Sichuan Birth Defects Clinical Research Center, West China Second University Hospital, Sichuan University, Chengdu, China

**Keywords:** adenovirus, respiratory infections, proteomics, E-selectin, children

## Abstract

**Background:**

Human adenoviruses are prevalent pathogens that cause severe acute respiratory infections. The clinical presentation of the adenoviral pneumonia is varied; in severe cases, they may cause systemic multi-system damages. Currently, early clinical differential diagnosis is difficult under the existing testing methods, the study identified potential biomarkers by screening and validating differentially expressed proteins (DEPs), and aimed at distinguishing between severe and non-severe adenovirus pneumonia in children aged <14 years.

**Methods:**

DEPs were identified using data-independent acquisition (DIA) quantitative proteomics technology, and potential biomarkers were further validated using an enzyme-linked immunosorbent assay (ELISA).

**Results:**

Twenty-seven identical DEPs were found in patients with severe adenovirus pneumonia. Among these, 10 were downregulated, and 17 were upregulated. In the protein–protein interaction network, five proteins were located at the center of the functional network. Among these, E-selectin showed significantly higher serum expression levels in the severe adenoviral pneumonia group than in adenoviral pneumonia and control groups (*p* < 0.001). ELISA results were consistent with the proteomic analyses. The receiver operating characteristic (ROC) curve for E-selectin revealed a sensitivity of 79.31% and a specificity of 96.55%, with an area under the curve (AUC) of 0.92.

**Conclusion:**

E-selectin has potential as a novel biomarker for severe adenoviral pneumonia, and offers insights for improved diagnosis and clinical management.

## 1 Introduction

Human adenovirus is a common pathogen that causes acute severe respiratory infections, with global prevalence among respiratory viruses. Its detection rate ranges from 4 to 10% (1, 2) and can reach up to 20% in some countries (3, 4). Approximately one-third of adenoviral pneumonia cases progress to severe pneumonia (5), resulting in the highest morbidity and mortality rates for viral pneumonia in children under 5 years of age (6). Consequently, adenoviral pneumonia has become a major focus in pediatric clinical practice.

Despite significant advances in understanding the molecular mechanisms of adenoviral lung infections, effective diagnostic methods remain limited. Traditional diagnostic methods, such as imaging and pathogen detection, may not always provide adequate information or accurately predict disease progression. Studies have attempted early identification of severe pneumonia by considering factors including patient age, clinical signs (e.g., fever duration, respiratory rate), and conventional laboratory tests. However, these studies are often limited by single-center designs, small sample sizes, inconsistent data, and low practical applicability (7, 8).

A systematic review and meta-analysis of 17 high-quality studies involving 3,192 children examined biomarkers that could indicate pneumonia severity in children. The results suggested that the CRP, IL-6, IL-8, and procalcitonin (PCT) levels may help predict the progression to severe pneumonia in children (9). However, significant variation in mean values across these studies prevented the establishment of specific cut-off values. Therefore, high-quality meta-analyses or large-sample studies are warranted to validate these findings.

It is crucial to identify new biomarkers to improve the existing diagnostic methods for severe adenovirus pneumonia. This study used data-independent acquisition (DIA) mass spectrometry for relative quantitative proteomic analysis to identify DEPs in patients with severe adenovirus pneumonia group than in the healthy controls and patients with adenovirus pneumonia. E-selectin, a key protein of DEPs, was further verified using enzyme-linked immunosorbent assay (ELISA), and its sensitivity and specificity were analyzed using receiver operating characteristic (ROC) curve analyses. This study provides a valuable candidate serum marker for evaluating and diagnosing severe adenovirus pneumonia.

## 2 Materials and methods

### 2.1 General information

Patients diagnosed with adenovirus pneumonia and admitted to West China Second University Hospital, Sichuan University, from March 1, 2023, to March 1, 2024, were selected for this study. Eighty-seven cases were enrolled, including 29 healthy controls, 29 patients with non-severe adenoviral pneumonia, and 29 patients with severe adenoviral pneumonia. The study protocol was approved by the Ethics Committee of the West China Second University Hospital, and informed consent was obtained from the patients' parents or legal guardians.

### 2.2 Diagnostic criteria for adenovirus pneumonia

Diagnosis was based on the technical guidelines for the prevention and treatment of human adenovirus (HAdV) respiratory infections and the treatment protocol for pediatric adenovirus pneumonia (10, 11). Patients meeting the diagnostic criteria for pneumonia and adenovirus infection were confirmed through an adenovirus infection test. Nasopharyngeal swabs, sputum, and bronchoalveolar lavage fluid samples were collected, and real-time quantitative polymerase chain reaction (qRT-PCR) was used to detect adenovirus nucleic acid. A viral load > 103 copies/mL in any of the above specimens confirmed adenovirus infection.

### 2.3 Inclusion and exclusion criteria

The affected children were divided into non-severe adenovirus pneumonia and severe adenovirus pneumonia groups based on their condition and according to the 2019 guidelines for the management of community-acquired pneumonia in children (12). The inclusion criteria for patients with adenovirus pneumonia in this study were as follows: (a) age ≥ 28 days to <14 years, (b) diagnosed with pneumonia, and (c) positive for adenovirus nucleic acid. Patients were categorized into the severe pneumonia group if they met the pneumonia diagnostic criteria along with any of the following conditions: poor general condition, signs of dehydration or refusal to eat, unconsciousness, cyanosis, rapid respiratory rate (≥70 breaths/min in infants and ≥50 breaths/min in children older than 1 year), respiratory distress (grunting, nasal flaring, and intercostal retractions), intermittent respiratory pauses, pulse oxygen saturation <92%, severe chest imaging findings (pulmonary collapse, multilobed lungs, pleural effusion, pneumothorax, lung abscess, or lung necrosis), or extrapulmonary complications. The exclusion criteria were as follows: children with tuberculosis, non-infectious lower respiratory tract infections, autoimmune diseases, concomitant asthma or chronic respiratory infections, blood disorders or congenital genetic diseases, or incomplete data.

### 2.4 Data-independent acquisition relative quantitative proteomics

The high-abundance proteins in the serum were removed using the High-Selective TM Top14 Abundant Protein Depletion Resin kit, and the remaining proteins were concentrated to an appropriate volume. Subsequently, the proteins were digested with trypsin, and the peptides were quantified using the Thermo Fisher Scientific Peptide Quantification Kit. Based on the peptide quantification results, the peptides were re-dissolved in a spectrometry loading buffer containing appropriate iRT peptides, which were used to calibrate the retention time, and analyzed using an EASY-nLC system (Thermo, USA) coupled with a timsTOF Pro2 mass spectrometer (Bruker, Germany) at Majorbio Bio-Pharm Technology Co. Ltd. (Shanghai, China). Data-independent acquisition (DIA) data were acquired using the timsTOF Pro2 mass spectrometer operated in DIA-PASEF mode. Spectronaut software (Version 14) was used to search the DIA-PASEF raw data. Retention times were corrected using iRT, and six peptides per protein and three daughter ions per peptide were selected for quantitative analysis.

### 2.5 Bioinformatics analysis

Bioinformatics analysis of proteomic data was performed using the Majorbio Cloud platform (https://cloud.majorbio.com). *P*-values and fold changes (FC) for the proteins between the two groups were calculated using the R package “*t*-test.” The thresholds of fold change (>1.2 or <0.83) and *p*-value <0.05 were used to identify DEPs. Functional annotation of all identified proteins was performed using Gene Ontology (GO; http://geneontology.org/) and Kyoto Encyclopedia of Genes and Genomes (KEGG) pathways (http://www.genome.jp/kegg/). The DEPs were further used for GO and KEGG enrichment analyses. Protein–protein interaction analysis was performed using String v11.5.

### 2.6 ELISA assay

The human E-selectin ELISA kit was purchased from R&D Systems and the experiments were performed according to the manufacturer's instructions. This was used to measure the protein concentration in each serum sample in the validation set.

### 2.7 Statistical analyses

The data were analyzed using SPSS Statistics 19 (SPSS, Inc., Chicago, IL). Experimental data conforming to a normal distribution were presented as mean ± standard deviation, and comparisons between groups were made using a 2-sample *t*-test. Measurement data that were not normally distributed were represented as median (M) and interquartile range (P25, P75), with comparisons between groups conducted using the Mann–Whitney *U*-test. Enumeration data were expressed as cases (%) and comparisons between groups were performed using Pearson's chi-squared test. The prediction of risk factors was investigated through binary logistic regression analysis combined with the ROC curve. A *p*-value of <0.05 was considered statistically significant.

## 3 Result

### 3.1 Characteristics of the study population

The demographic and clinical characteristics of the study population are shown in Table 1. No significant difference was observed in age and gender among the groups (*p* > 0.05). In the non-severe group, 18 cases (62.1%) were males and 11 cases (37.9%) were females. In the severe group, 17 cases (58.6%) were males and 12 cases (41.4%) were females. The median age was 32 months in the non-severe group and 30 months in the severe group (Table 1). Among the 58 cases, 53 (91.2%) had fever after being infected with adenovirus. The severe group had significantly more wheezing than the non-severe group (*p* < 0.05; Table 1). The electrolyte disturbance was significantly higher in the severe group than in the non-severe group (*p* < 0.05). The incidence of children with severe pneumonia combined with two or more co-morbidities was significantly higher than that in the non-severe disease group (*p* < 0.05; Table 1).

**Table 1:** The demographic and clinical characteristics of the study population.

**Variables**	**Healthy controls (*n* = 29)**	**Non-severe (*n* = 29)**	**Severe (*n* = 29)**	***P*-value**
Gender (male/female), *N*	16/13	18/11	17/12	*P* > 0.05
Age (months)	24 (19, 35)	32 (16, 36)	30 (20, 42)	*P* > 0.05
**Symptoms**, ***N*** **(% of total samples)**				
Fever	/	25 (86.2)	28 (96.6)	0.16
Wheezing	/	2 (6.9)	9 (31.0)	0.02
Rales	/	24 (82.7)	25 (86.2)	0.72
Respiratory failure	/	0 (0)	2 (6.9)	0.15
Liver injury	/	0 (0)	2 (6.9)	0.15
	/	1 (3.4)	2 (6.9)	0.55
Electrolyte disturbances	/	0 (0)	5 (17.2)	0.02
Vomiting	/	4 (13.8)	5 (17.2)	0.72
Diarrhea	/	3 (10.3)	4 (13.8)	0.69
Coagulation disorders	/	0 (0)	3 (10.3)	0.07
Toxic encephalopathy	/	0 (0)	1 (3.4)	0.31
With two or more comorbidities	/	1(3.4)	6 (20.7)	0.04

The age data are presented as median (P25, P75). Severe: Children diagnosed as severe adenovirus pneumonia; Non-severe: Children diagnosed as adenovirus pneumonia.

### 3.2 Differentially expressed proteins

**Figure 1:**
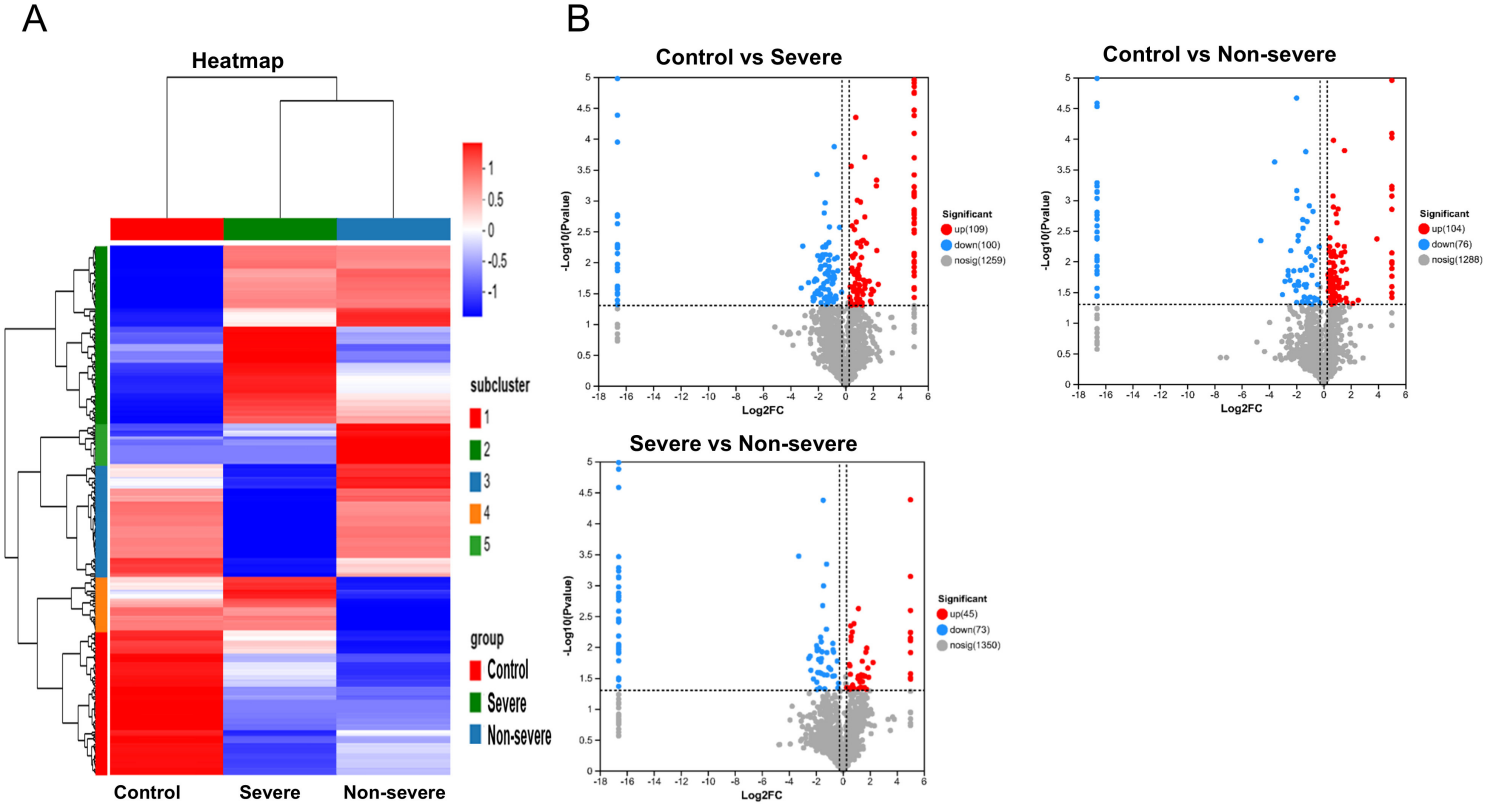
The serum proteomics displayed differential expression of proteins in children with adenovirus pneumonia as compared to healthy control. **(A)** The heatmap of differentially expression proteins in different groups. **(B)** The volcano map of differentially expression proteins in different groups. Upregulated proteins are shown by red, downregulated by blue. Control: healthy children. Severe: Children diagnosed as severe adenovirus pneumonia; Non-severe: Children diagnosed as adenovirus pneumonia.

Nine cases existed (three cases were randomly selected from each group) that underwent DIA-relative quantitative proteomics in this study. The quantification results of the identified proteins were normalized using the total peak area ratio for differential protein screening and statistical analyses. The test function in R was used to calculate the significance *p*-value and fold change in the differences between groups. Proteins with a significance test *p*-value <0.05 and a fold change >1.2 were considered DEPs. Overall, 209 DEPs were observed between the severe adenoviral pneumonia and the normal control groups, of which 109 were upregulated and 100 were downregulated. Moreover, 280 DEPs occurred between the non-severe adenovirus pneumonia and normal control groups, of which 104 were upregulated and 76 were downregulated. Additionally, 118 DEPs were observed between the severe and non-severe adenovirus pneumonia groups, of which 45 were upregulated and 73 were downregulated (Figures 1A, B). A comparison of the DEPs of the severe adenovirus pneumonia group vs. the control group and the severe adenovirus pneumonia group vs. the non-severe adenovirus pneumonia group revealed 27 identical DEPs. Among these, 10 were downregulated and 17 were upregulated. Table 2 summarizes information on the DEPs.

**Table 2:** The DEPs in severe adenovirus pneumonia group that compared with non-severe adenovirus pneumonia group and healthy controls.

**Accession**	***P*-value**	**FDR**	**Regulate**
A0A0S2Z3V0	6.98E-05	0.000943	Down
A0A7S5C3F5	0.00042	0.003783	Down
A0A4P8J422	0.000626	0.004225	Down
B0QY93	0.00203	0.00699	Down
A0A5C2G2M2	0.002565	0.00699	Down
A0A5C2G8M0	0.002883	0.007076	Down
O75909	0.005587	0.01256	Down
A0A8V8TND7	0.00771	0.014869	Down
A0A5C2GTJ7	0.014254	0.020255	Down
A0A7S5BYD1	0.020293	0.023823	Down
C9JX88	3.25E-07	8.78E-06	Up
A8K3C3	0.000841	0.004542	Up
SELE	0.001242	0.005587	Up
D3DPK5	0.002353	0.00699	Up
A0A3B3IRX2	0.002589	0.00699	Up
A0A024RDY2	0.006048	0.01256	Up
Q14314	0.008736	0.015724	Up
A0A7S5C1W5	0.010147	0.017123	Up
A0A7I2V2U8	0.010823	0.017189	Up
A0A5C2FT71	0.011643	0.017465	Up
C9JL85	0.018233	0.023823	Up
A0A7S5BYP1	0.018766	0.023823	Up
Q05315	0.019694	0.023823	Up
P62328	0.022378	0.024341	Up
B4DDD6	0.02281	0.024341	Up
A0A024R0A1	0.023439	0.024341	Up
B4DGL0	0.025638	0.025638	Up

### 3.3 The DEPs were functionally annotated by GO and KEGG

**Figure 2:**
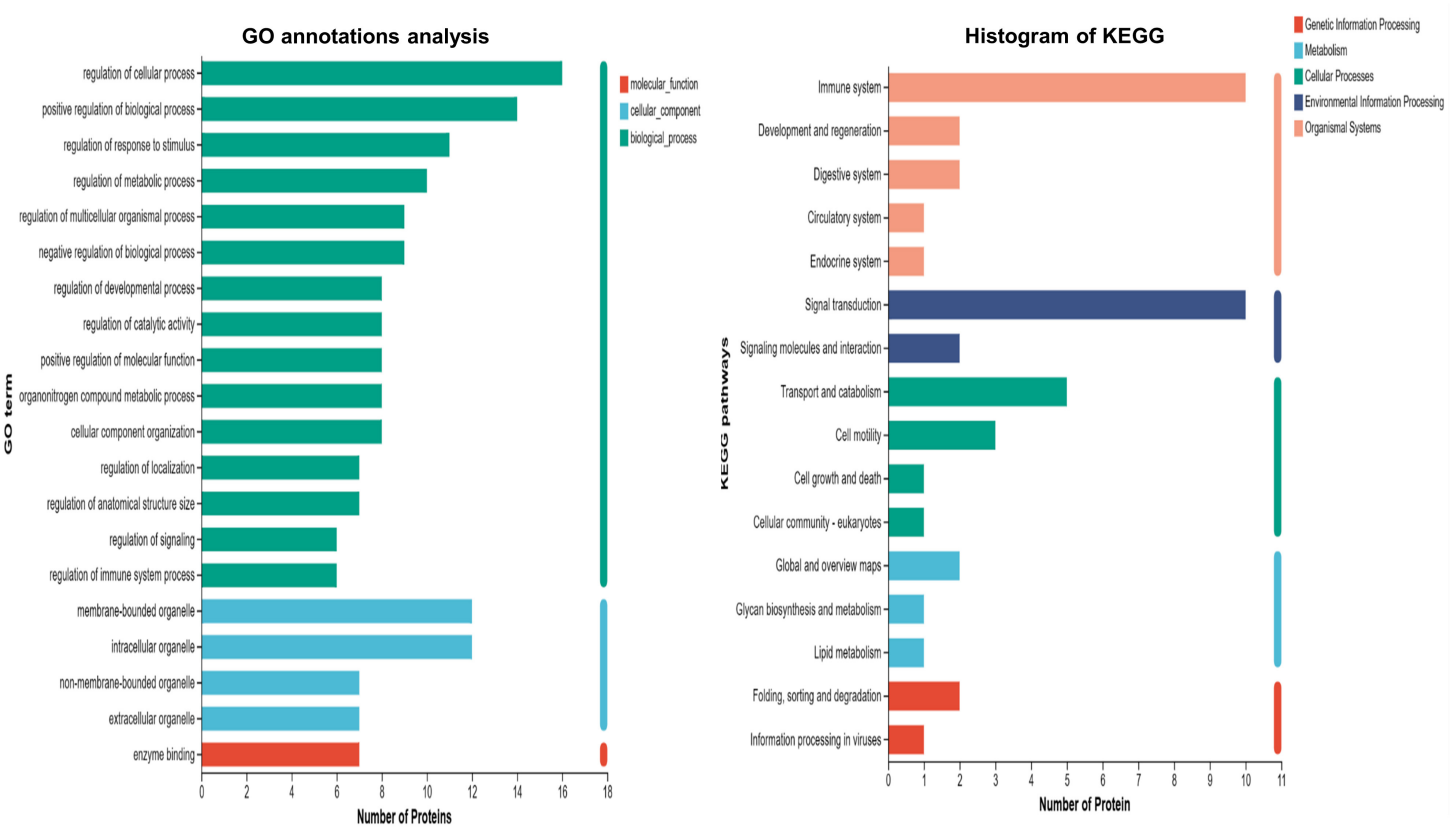
GO and KEGG annotation analysis of differentially expression proteins in severe adenovirus pneumonia group with the healthy control and non-severe adenovirus pneumonia group but showed no differences within these two groups. The horizontal coordinate is the number of proteins and the vertical coordinate is the annotated classification.

GO and KEGG functional annotations were performed on proteins that showed expression differences when comparing the severe adenovirus pneumonia group with the control and non-severe adenovirus pneumonia groups but showed no differences between the latter two groups. The results revealed that most DEPs were distributed intracellularly and within cell membranes. Biological process analysis revealed that most DEPs were involved in cellular and biological processes, regulation of responses to stimuli, metabolic and multicellular organism regulation, negative regulation of biological processes, positive regulation of molecular functions, catalytic activity regulation, immune system, and signal transduction regulation. Molecular function annotation indicated that the DEPs were mainly related to enzyme binding. KEGG functional annotation revealed that the DEPs were primarily involved in the immune system and signal transduction pathways (Figure 2).

### 3.4 GO and KEGG enrichment analysis of DEPs

**Figure 3:**
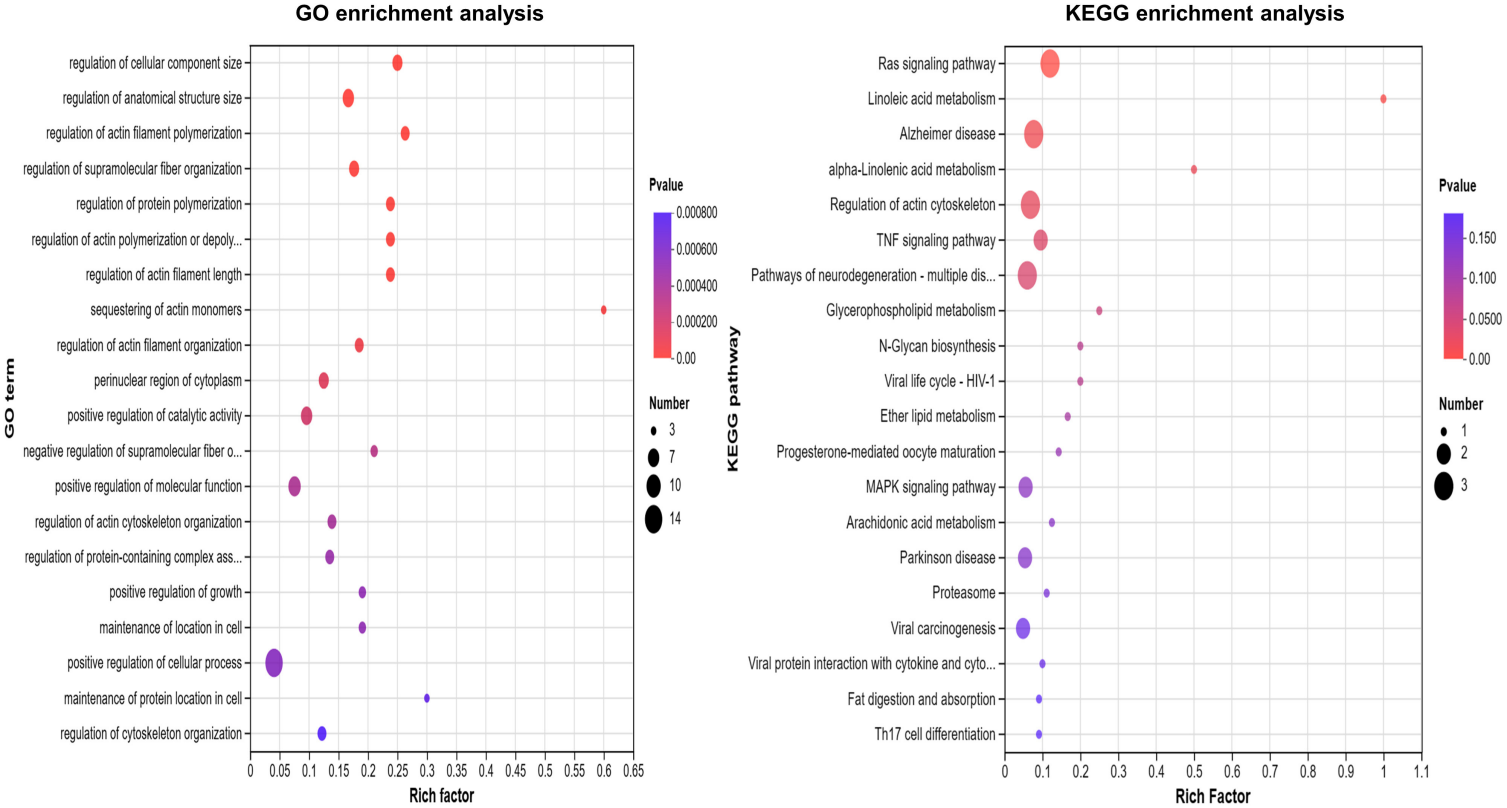
GO and KEGG enrichment analysis of differentially expression in severe adenovirus pneumonia group. The larger the bubble, the more protein there is. The color of the bubbles indicates the *p*-value, the smaller the *p*-value, the greater the significance.

The top 10 GO terms among these DEPs included the regulation of cell component size, anatomical structure size, actin filament polymerization, supramolecular fiber organization, actin filament length, actin polymerization or depolymerization, protein monomer depolymerization, actin monomer depolymerization, positive regulation of actin filament organization, and catalytic activity. The main signaling pathways involving these DEPs included the Ras signaling pathway, linoleic acid metabolism, alpha-linolenic acid metabolism in Alzheimer's disease, actin cytoskeleton regulation, and the TNF signaling pathway (Figure 3).

### 3.5 Differentially expressed protein interaction network analysis

**Figure 4:**
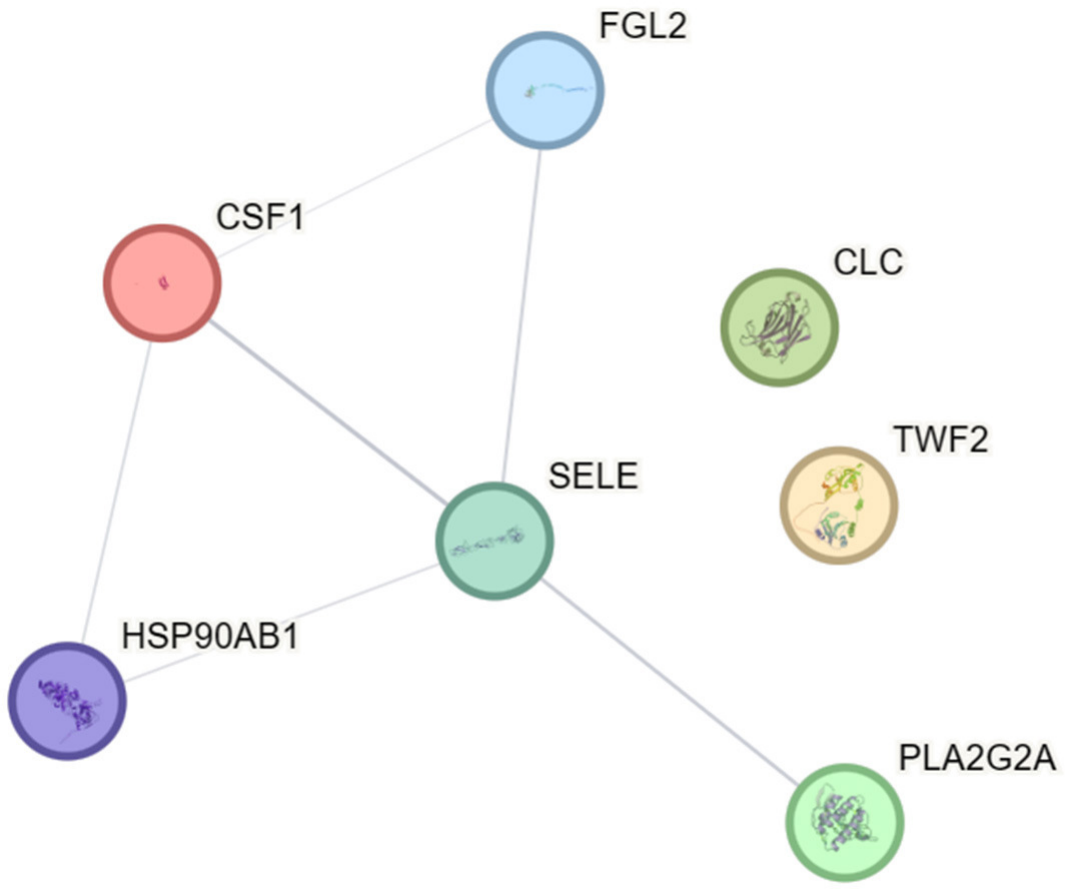
Interaction diagram of DEPs in severe adenovirus pneumonia group.

STRING software analysis of the protein–protein interaction network revealed key interacting proteins among the DEPs, including SELE, CSF1, FGL2, HSP90AB1, and PLA2G2A (Figure 4). SELE (known as E-selectin) was selected for validation experiments because (1) limited research exists on E-selectin in adenoviral pneumonia; (2) E-selectin commercial kits are available; (3) it shows a high differential fold change; and (4) bioinformatics analysis indicates that E-selectin plays a critical role in certain biological functions.

### 3.6 Serum concentrations of E-selectin were examined by ELISA

**Figure 5:**
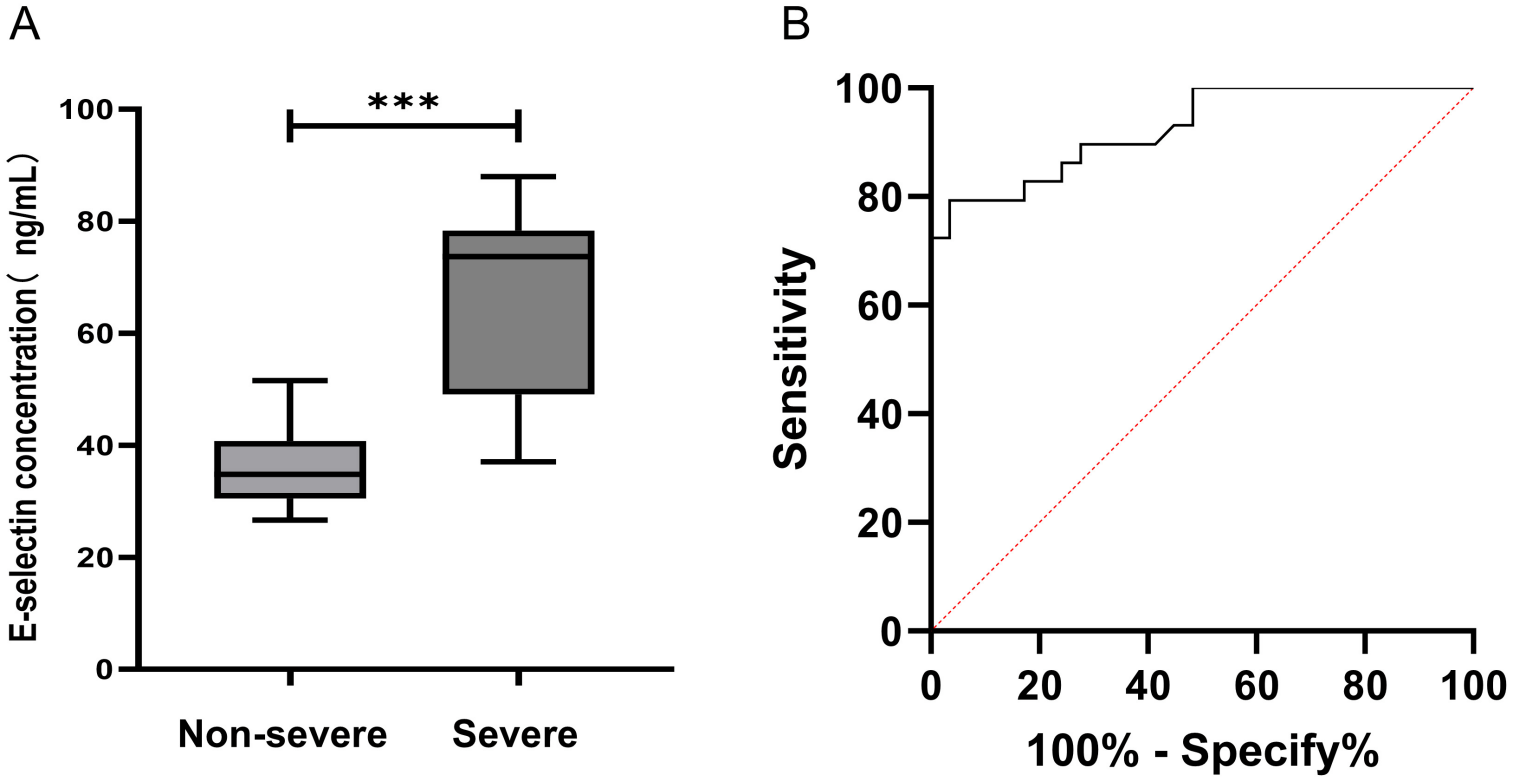
Analysis of E-selectin in serum. **(A)** The serum levels of E-selectin were measured by ELISA. Non-severe adenovirus pneumonia group (*n* = 29), severe adenovirus pneumonia group (*n* = 29). Median values were shown by a horizontal line. **(B)** ROC curve analysis. ROC curves analysis of E-selectin to discriminate severe adenovirus pneumonia from non-severe adenovirus pneumonia. Severe: Children diagnosed as severe adenovirus pneumonia; Non-severe: Children diagnosed as non-severe adenovirus pneumonia. ***p < 0.001.

The serum concentration of E-selectin in the non-severe adenovirus pneumonia group and the severe adenovirus pneumonia group were 36.37 ± 6.68 and 65.89 ± 16.59 ng/mL, respectively (Figure 5A). The result revealed that the serum level of E-selectin in the severe adenovirus pneumonia group was significantly higher than that in the non-severe adenovirus pneumonia group (*p* < 0.001). The ELISA results for E-selectin were consistent with the proteomic profiling. A diagnostic model was established to analyze the serum concentration data of E-selectin. The results showed that the AUC and ROC curve threshold values for E-selectin were 0.92 and 48.62 ng/mL, respectively. The sensitivity and specificity of E-selectin diagnosing severe adenovirus pneumonia were 79.31 and 96.55%, respectively (Figure 5B).

## 4 Discussion

Adenovirus pneumonia is among the viral pneumonia with the highest morbidity and mortality rates in children under 5 years of age worldwide (13–15). Currently, early identification and diagnosis of severe adenovirus pneumonia remain difficult. The composition and quantity of low-abundance proteins changed significantly in the sera of children infected with adenoviruses. Therefore, exploring the role of these proteins in the progression of adenoviral pneumonia is crucial for its diagnosis, treatment, and monitoring. Proteomics can detect and compare the proteins expressed in the serum between disease and control groups, screen out proteins whose expression levels change significantly, and further explore their structural and functional characteristics. This provides a new method to study the pathogenesis of diseases, identify potential biological markers for disease diagnosis, and identify drug targets for disease treatment (16–18).

This study applied DAI proteomics technology to investigate changes in serum protein expression profiles in children infected with adenovirus, focusing on the proteins that exhibited significant differences in expression between severe adenovirus pneumonia, non-severe adenovirus pneumonia, and control groups. A total of 27 DEPs were identified in this study. These proteins may be closely related to the severity of adenovirus pneumonia. GO analysis clarified the biological significance of these 27 DEPs. The CC analysis by GO revealed that most DEPs are distributed intracellularly and in the cell membrane, indicating they are signal transduction proteins. It plays an important role in mediating extracellular signal to intracellular signal transduction in host defense and inflammation development caused by adenovirus infection. BP analysis showed that proteins related to modulation take a large proportion. Furthermore, the GO enrichment analysis revealed that DEPs were primarily involved in the assembly and regulation of actin and participated in the signaling pathways of the actin cytoskeleton. Previous studies have indicated that severe pneumonia affects the cell structure across multiple layers and aspects. Severe pneumonia can cause endoplasmic reticulum (ER) stress, resulting in protein misfolding and an unfolded protein response. Inflammatory mediators and pathogens can disrupt the cytoskeleton, causing changes in cell morphology, loss of motility, and impaired cell division. Tight and adhesive junctions between epithelial and endothelial cells play key roles in maintaining tissue structure and function. The inflammatory response caused by severe pneumonia can disrupt these cell junctions, leading to alveolar barrier dysfunction, increased lung permeability, pulmonary edema, and acute respiratory distress syndrome (19–21). The differential proteins found in this study suggest that cell structure damage and disruption could be one of the causes of the severe clinical symptoms of severe adenovirus pneumonia.

We further explored which signaling pathways were involved in the regulation of DEPs through KEGG functional annotations. This study revealed that those DEPs were predominantly involved in the Ras signaling pathway. It is well-known that the Ras signaling pathway plays a crucial role in various cellular processes, including cell growth, morphological transformation, stress response, and cell death. It also plays an important role in viral infections. The Ras pathway plays a key role in the regulation of *Candida albicans* (22). Kaposi's sarcoma-associated herpes virus and Epstein-Barr virus promote the survival and proliferation of host-infected cells through the Ras signaling pathway (23). In addition, reoviruses promote viral protein synthesis by activating the Ras pathway in host cells (24). Few studies have investigated the mechanisms underlying the Ras pathway in adenovirus-induced respiratory infections. Further research is needed to determine the precise regulatory mechanisms and the specific cell types that are affected.

Simultaneously, the functional network analysis of DEPs showed that E-selectin proteins appeared in the center of the intersection of functional networks. In this study, STRING database analysis revealed that E-selectin is a key protein in the protein-protein interaction network. The results of ELISA in this study showed that the serum E-selectin level in patients with severe adenovirus pneumonia was significantly higher than that in the non-severe adenovirus pneumonia group. This result is consistent with the results of DAI proteomics. E-selectin is mainly expressed in activated endothelial cells, serves as a marker of endothelial dysfunction, inflammation, and injury, and is critical for attracting immune effector cells to inflammatory sites. It contributes to the development of numerous acute and chronic inflammatory conditions (25, 26). E-selectin plays an important role in inflammatory diseases, including vascular (27) and renal inflammation (28). Several studies on Coronavirus Disease 2019 (COVID-19) have identified E-selectin as a marker of severe COVID-19, with serum E-selectin concentrations being positively correlated with disease severity (29–31). In studies of prognostic factors in hospitalized patients with acute inflammatory respiratory diseases, serum E-selectin levels were significantly higher in deceased patients than in survivors, with serum E-selectin levels serving as independent predictors of prognosis (32).

Presently, few studies exist on the biomarkers of severe pneumonia caused by adenovirus infection in children. This study revealed that E-selectin has high sensitivity and specificity for the diagnosis of severe adenovirus pneumonia, suggesting that it may be a potential biological marker.

In summary, this study utilized proteomics technology to analyze serum proteins in children with adenovirus pneumonia, revealing that E-selectin could potentially serve as a biological marker for the diagnosis of severe adenovirus pneumonia with high sensitivity and specificity. However, this study had the following limitations: First, it was a single-center study with relatively small sample sizes in each group. Therefore, future research should focus on expanding the sample size and conducting multicenter, blinded trials to further assess the marker's validity and reliability. Second, this study excluded cases with co-infections, such as multiple viral infections or viral and bacterial co-infections. Future research should consider these factors to fully understand the behavior of E-selectin in different infection contexts.

## 5 Conclusion

Our study analyzed the plasma proteome profile of children with adenovirus pneumonia and identified 27 DEPs between severe adenovirus pneumonia, non-severe adenovirus pneumonia, and healthy control groups. It was revealed that E-selectin has potential value as a serum biomarker for diagnosing severe adenovirus pneumonia. Future studies should focus on validating these findings across diverse populations and exploring the clinical applications of E-selectin to improve the management and prognosis of children with severe adenoviral pneumonia.

## Data Availability

The datasets presented in this study can be found in the Integrated Proteome Resources under accession number IPX0010173000 (https://www.iprox.cn/page/SSV024.html;url=1731033314176NWt3). Further inquiries can be directed to the corresponding author.
